# Kynurenic acid, an aryl hydrocarbon receptor ligand, is elevated in serum of Zucker fatty rats

**Published:** 2016-08-29

**Authors:** G Oxenkrug, J Cornicelli, M van der Hart, J Roeser, P Summergrad

**Affiliations:** 1 Department of Psychiatry, Tufts University School of Medicine, Boston, USA; 2 Charles River, Inc., USA; 3 Brains On-Line, S. San Francisco, USA

**Keywords:** zucker fatty rats, kynurenic acid, aryl hydrocarbon receptor, obesity, metabolic syndrome

## Abstract

Obesity is an increasingly urgent global problem and the molecular mechanisms of obesity are not fully understood. Dysregulation of the tryptophan (Trp) – kynurenine (Kyn) metabolic pathway (TKP) have been suggested as a mechanism of obesity and described in obese humans and in animal models of obesity. However, to the best of our knowledge, TKP metabolism has not been studied in leptin-receptor-deficient Zucker fatty rats (ZFR) (*fa/fa*), the best-known and most widely used rat model of obesity. We were interested to determine if there are any deviations of TKP in ZFR. Concentrations of major TKP metabolites were evaluated (HPLC- MS method) in serum of ZFR (fa/fa) and age-matched lean rats (*FA/-*). Concentrations of kynurenic acid (KYNA) were 50% higher in ZFR than in lean rats (p<0.004, Mann-Whitney two-tailed test). Anthranilic acid (AA) concentrations, while elevated by 33%, did not reach statistical significance (p<0.04, one-tailed test). Elevated KYNA serum concentrations might contribute to development of obesity via KYNA-induced activation of aryl hydrocarbon receptor. Present results warrant further studies of KYNA and AA in ZFR and other animal models of obesity.

## Introduction

**Figure 1:**
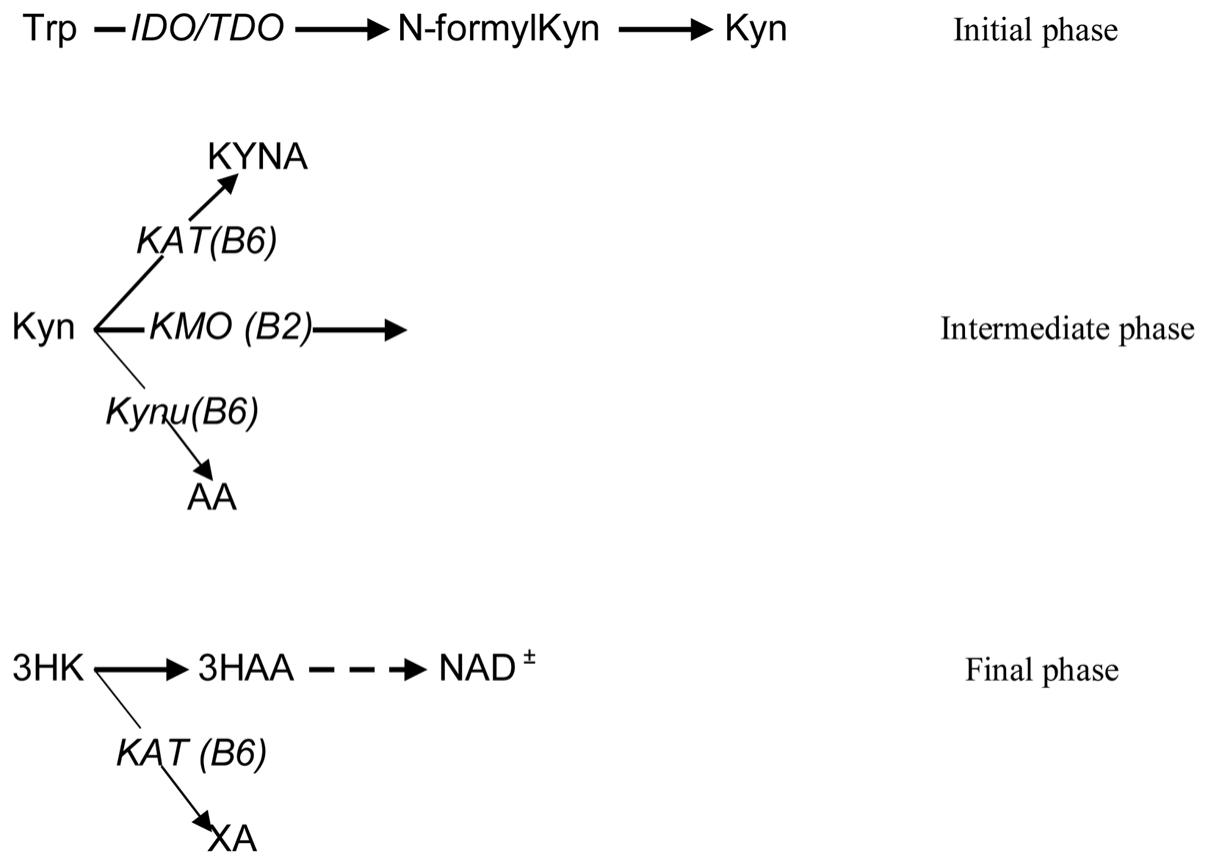
Three major phases of Tryptophan – Kynurenine metabolic pathway. Abbreviations: Trp: tryptophan; Kyn: kynurenine; KYNA: kynurenic acid; 3HK: 3-hydroxykynurenine; AA: anthranilic acid; 3HAA: 3-hydroxyanthranilic acid; XA: xanthurenic acid (3-hydroxyKYNA); NAD^+^: nicotinamid adenine dinucleotide; KMO: kynurenine 3-monooxygenase; Kynu: kynureninase; KAT: kynurenine aminotransferase 2

Obesity is an increasingly urgent global problem. Molecular mechanisms of obesity have not been fully understood. Dysregulation of tryptophan (Trp) – kynurenine (Kyn) metabolic pathway (TKP) have been suggested to contribute to the development of obesity [1,2]. In mammals, TKP consists of three major phases: the initial phase in which conversion of Trp into KYN (via N-formylKYN) is catalyzed by inflammation-induced indoleamine-2,3-dioxygenase 1 (*IDO*) or stress-activated Trp-2,3-dioxygenase 2 (*TDO*); the intermediate phase characterized by Kyn conversion into 3-hydroxykynurenine (3-HK), kynurenic (KYNA) and anthranilic (AA) acids; and final phase in which production of NAD^+^ is initiated by 3-HK conversion into 3-hydroxyAA (Figure 1) [3]. KYNA and AA are the end-products of KTP in astrocytes and adipocytes because Kyn-3-monooxygenase (*KMO*), a riboflavin (vitamin B2)-dependent enzyme, that catalyzes Kyn conversion into 3-HK, is not expressed in these tissues [4,5].

Activation of TKP initial phase was reported in animal models of obesity [6,7] and in obese human subjects [8–10]. Recent studies found different patterns of TKP dysregulation in obese mice and in human obesity and concluded that these mouse models [high-fat diet induced-obesity and the leptin-deficiency (ob/ob)] are inappropriate for studies of TKP involvement in mechanisms of human obesity [11]. However, to the best of our knowledge, TKP metabolism has not been studied in leptin-receptor-deficient Zucker fatty rats (ZFR) (*fa/fa*), the best-known and most widely used rat model of obesity. We were interested to determine if there are any abberations of TKP in ZFR by comparing serum concentrations of major TKP metabolites in ZFR (fa/fa) and lean rats (*FA/-)*.

## Methods

Serum samples (drawn after 5 hrs of fasting) from male ZFR (fa/fa) and lean (*FA/-*) rats (6 – 8 weeks of age) were provided by Charles River, Inc. and stored at -50°C until analysis. Trp, Kyn, KYNA, AA, 3-HK, and XA were analyzed by modified HPLC – MS method [12,13].

### Statistical analysis

Results are presented as mean ± standard error (Trp and Kyn in μM and AA, KYNA, 3-HK and XA in nM). Statistical significance of differences between lean and ZFR (six rats in each group) was assessed by Mann-Whitney test.

## Results

### Initial phase of TKP

There were no difference of Trp and Kyn serum concentrations between ZFR and lean rats (Table 1).

Kyn:Trp ratio (an indirect marker of activity of enzymes catalyzing Trp conversion into Kyn) was not different between ZFR and lean rats (Table 1).

### Intermediate phase of TKP

Serum concentrations of KYNA were elevated (by 50%) in ZFR (Table 1). There was a strong tendency to elevation of AA concentrations (by 33%). 3-HK concentrations did not differ between ZFR and lean rats.

### Final phase of TKP

Concentrations of XA, a suggested diabetogenic 3-HK metabolite [17], did not differ between ZFR and lean rats.

## Discussion

Present finding of elevated concentrations of serum KYNA (and, probably, of AA) in ZFR is important because, to the best of our knowledge, it is the first recognition of KTP dysregulation in ZFR; specifically, dysregulation of the intermediate phase of KTP in ZFR.

Our finding is in line with reported positive correlation of serum KYNA concentrations with BMI in obese subjects [4,14].

Elevation of serum KYNA concentrations might result from up-regulated KYNA biosynthesis in fat tissue, liver and monocyte/macrophages. Formation of KYNA and AA from Kyn is catalyzed by the vitamin B6 (PLP)-dependent enzymes *KAT* and *Kynu*, respectively (Figure 1). However, since *KAT* and *Kynu* are substrate-unsaturated enzymes, they are able to process additional amount of Kyn created by *KMO* inhibition. Thus, KYNA and AA were elevated in brain, liver and plasma of *KMO*(−/−) mice [15]. Furthermore, increased formation of KYNA and AA without activation of *KAT* and *Kynu* was observed in baboons and mice fed vitamin B2 (but not vitamin B6) deficient diets [16,17]. Therefore, KYNA elevation in serum of ZFR may be a consequence of *KMO* deficiency in fat tissue that does not express *KMO* genes [4]. On the other hand, KYNA might be synthesized by resident macrophages infiltrating omental adipose tissue, as this has been observed in women with obesity [4]. Therefore, *KMO* inhibition and/or *Kynu* activation may contribute to our observation of elevated serum KYNA (and AA) in ZFR. Identification of origin of serum KYNA elevation in ZFR needs further studies.

Functional implications of elevated KYNA in ZFR could depend on KYNA antagonism to N-methyl-d-aspartate (NMDAR) and alpha7 nicotinic acetylcholine receptors (α7nAChR) and activation of aryl hydrocarbon receptor (AHR).

AHR regulates xenobiotic-metabolizing enzymes such as aryl hydrocarbon hydroxylase (cytochrome P450) in humans, mice, rats and neonatal (but not adults) rabbits [18]. AHR over-activation promoted [19,20] while AHR deficiency protected mice from diet-induced obesity [21]. TKP metabolites (Kyn, KYNA and XA) are the endogenous human AHR ligands with potency comparable to exogenous ligands (e.g., 2,3,7,8-tetrachlorodibenzo-p-dioxin) [22,23]. Aryl hydrocarbon hydroxylase (cytochrome P450) is the major enzyme induced under control of the AHR [18]. Although we are not aware of AHR evaluation in ZFR, deficiency of AHR signaling pathways might be suggested by lower activity of hepatic microsomal aryl hydrocarbon hydroxylase and lower nuclear transcription rate of CYP2B1/2B2 mRNA in ZFR than in lean rats [24,25]. Up-regulated formation of KYNA, one of the strongest human hepatic AHR ligands [22], may represent an adaptive response aimed to overcome impairment of AHR signaling pathways in ZFR.

Besides interaction with AHR, elevated KYNA might affect ZFR via antagonism to NMDAR and α7nAChR. It was suggested that enhanced production of KYNA in astrocytes and increased extracellular KYNA inhibit dopamine (DA) release by blocking α7nAChR [26]. Decreased D2 receptor binding and elevated D2/3 receptor availability were reported in obesity [27,28]. Impaired DA function in ZFR was considered to be acquired, rather than inherited, trait caused by circulating factors associated with obesity [29]. Our present data suggest that elevated serum KYNA might be one of such circulating factors.

Present results warrant further studies of KYNA and AA in the ZFR as a translatable model for assessing the impact of changing this metabolic pathway on obesity and its comorbid conditions.

## Figures and Tables

**Table 1:** Kynurenines (serum) concentrations in Zucker fatty and lean rats.

N=6	Lean	Obese	P*	P**
Trp (μM)	133.60 ± 6.64	116.80 ± 4.14	ns	
Kyn (μM)	1.89 ± 0.14	1.87 ± 0.16	ns	
AA (nM)	60.08 ± 6. 74	80.96 ± 15.55	0.08	0.04**
KYNA (nM)	70.06 ± 2.77	105.05 ± 9.98	0.004*	0.002**
3HK (nM)	19.23 ± 0.98	20.57 ± 1.84	ns	
XA (nM)	13.10 ± 1.50	14.21 ± 1.54	ns	
Kyn/Trp	1.41 ± 0.09	1.60 ± 1.01	ns	

P* - Mann-Whitney: P* - two-tailed test; P** - one-tailed test.
